# Supplementary material for: Dietary Intake, Eating Behavior, Physical Activity, and Quality of Life in Infertile Women with PCOS and Obesity Compared with Non-PCOS Obese Controls
Source: Nutrients. 2021 Oct 8;13(10):3526. doi: 10.3390/nu13103526 (PMC8538395; doi:10.3390/nu13103526)
Supplement: Supplementary file 1 [file nutrients-13-03526-s001.zip › nutrients-1394804-supplementary.pdf]

Table S1. Analyses adjusting for confounders one at a time.

	age		waist-hip circumference ratio		HOMA-IR	
	Adjusted B or OR (95% CI)	p-value	Adjusted B or OR (95% CI)	p-value	Adjusted B or OR (95% CI)	p-value
Vegetable intake (g/day)	–8.64 (–23.33 to 6.05)	0.25	–16.3 (–30.7 to –1.85)	0.03	–16.6 (–32.8 to –0.50)	0.04
Fruit intake (g/day)	14.3 (–1.06 to 29.7)	0.07	7.81 (–7.29 to 22.9)	0.31	13.3 (–2.85 to 29.5)	0.11
Sugary drinks (glasses/day)						
< 0.5	ref	ref	ref	ref	ref	ref
0.5 – 1	1.00 (0.54 to 1.84)	1.00	1.15 (0.64 to 2.08)	0.64	0.94 (0.50 to 1.79)	0.86
> 1	1.10 (0.69 to 1.77)	0.67	1.48 (0.94 to 2.31)	0.09	1.02 (0.63 to 1.68)	0.93
Emotional eating overall sore	0.87 (–1.37 to 3.10)	0.45	0.69 (–1.46 to 2.84)	0.53	1.46 (–0.90 to 3.83)	0.23
Total moderate to vigorous physical activity (minute/week)						
< 200	ref	ref	ref	ref	ref	ref
200 – 700	1.26 (0.78 to 2.04)	0.35	1.15 (0.72 to 1.83)	1.15	1.50 (0.90 to 2.52)	0.12
> 700	0.95 (0.59 to 1.55)	0.84	1.02 (0.64 to 1.62)	0.93	1.05 (0.62 to 1.77)	0.87
Physical Component Score	–1.50 (–3.39 to 0.39)	0.12	–0.82 (–2.65 to 1.01)	0.38	–1.02 (–2.97 to 0.93)	0.30
Mental Component Score	1.17 (–0.83 to 3.16)	0.25	0.41 (–1.52 to 2.35)	0.67	0.68 (–1.41 to 2.77)	0.52
